# A multi-disciplinary commentary on preclinical research to investigate vascular contributions to dementia

**DOI:** 10.1016/j.cccb.2023.100189

**Published:** 2023-10-11

**Authors:** Sarmi Sri, Adam Greenstein, Alessandra Granata, Alex Collcutt, Angela C C Jochems, Barry W McColl, Blanca Díaz Castro, Caleb Webber, Carmen Arteaga Reyes, Catherine Hall, Catherine B Lawrence, Cheryl Hawkes, Chrysia-Maria Pegasiou-Davies, Claire Gibson, Colin L Crawford, Colin Smith, Denis Vivien, Fiona H McLean, Frances Wiseman, Gaia Brezzo, Giovanna Lalli, Harry A T Pritchard, Hugh S Markus, Isabel Bravo-Ferrer, Jade Taylor, James Leiper, Jason Berwick, Jian Gan, John Gallacher, Jonathan Moss, Jozien Goense, Letitia McMullan, Lorraine Work, Lowri Evans, Michael S Stringer, MLJ Ashford, Mohamed Abulfadl, Nina Conlon, Paresh Malhotra, Philip Bath, Rebecca Canter, Rosalind Brown, Selvi Ince, Silvia Anderle, Simon Young, Sophie Quick, Stefan Szymkowiak, Steve Hill, Stuart Allan, Tao Wang, Terry Quinn, Tessa Procter, Tracy D Farr, Xiangjun Zhao, Zhiyuan Yang, Atticus H Hainsworth, Joanna M Wardlaw

**Affiliations:** a UK Dementia Research Institute Headquarters, 6th Floor Maple House, London W1T 7NF, UK; b Division of Cardiovascular Sciences, The University of Manchester, Manchester M13 9PL, UK; c Geoffrey Jefferson Brain Research Centre, Manchester Academic Health Science Centre, University of Manchester, Manchester, UK; d Department of Clinical Neurosciences, Victor Phillip Dahdaleh Heart & Lung Research Institute, University of Cambridge, Papworth Road, Cambridge Biomedical Campus, Cambridge CB2 0BB, UK; e Centre for Clinical Brain Sciences, University of Edinburgh, Edinburgh, UK; f UK Dementia Research Institute Edinburgh, University of Edinburgh, Edinburgh, UK; g Centre for Discovery Brain Sciences, Chancellor's Building, The University of Edinburgh, Edinburgh, UK; h UK Dementia Research Institute Cardiff, Cardiff University, Cardiff CF24 4HQ, UK; i School of Psychology and Sussex Neuroscience, University of Sussex, Falmer, Brighton, East Sussex, UK; j Division of Neuroscience, School of Biological Sciences, Faculty of Biology, Medicine and Health, University of Manchester, Manchester, UK; k Biomedical and Life Sciences, Lancaster University, Lancaster, UK; l School of Psychology, University of Nottingham, Nottingham NG7 2UH, UK; m Physiopathology and Imaging of Neurological Disorders (PhIND), Normandie University, UNICAEN, INSERM UMR-S U1237, , GIP Cyceron, Institute Blood and Brain @ Caen-Normandie (BB@C), Caen, France; n Department of clinical research, Caen-Normandie University Hospital, Caen, France; o Division of Systems Medicine, School of Medicine, Ninewells Hospital & Medical School, University of Dundee, Dundee DD1 9SY, UK; p UK Dementia Research Institute, University College London, London WC1N 3BG, UK; q Stroke Research Group, Clinical Neurosciences, University of Cambridge, Cambridge CB2 0QQ, UK; r BHF Glasgow Cardiovascular Research Centre, Institute of Cardiovascular and Medical Sciences, University of Glasgow, Glasgow, UK; s Department of Psychology, University of Sheffield, Sheffield, UK; t Neuroscience Institute, University of Sheffield, Sheffield, UK; u Healthy Lifespan Institute, University of Sheffield, Sheffield, UK; v Department of Psychiatry, Warneford Hospital, University of Oxford, Oxford, UK; w Centre for Regenerative Medicine, Institute for Regeneration and Repair, The University of Edinburgh, Edinburgh, UK; x Neuroscience Program, University of Illinois, Urbana-Champaign, Urbana, IL, USA; y School of Cardiovascular & Metabolic Health, College of Medical, Veterinary & Life Sciences, University of Glasgow; Glasgow; UK; z Dementia Research Group, Department of Clinical Neurosciences, Bristol Medical School, University of Bristol, Bristol BS10 5NB, UK; aa Department of Brain Sciences, Imperial College London, London, UK; ab Department of Neurology, Imperial College Healthcare NHS Trust, London, UK; ac Stroke Trials Unit, University of Nottingham, Nottingham, UK; Stroke, Medicine Division, Nottingham University Hospitals NHS Trust, Nottingham, UK; ad Dementia Discovery Fund, SV Health Managers LLP, London, UK; ae Dementias Platform UK, Department of Psychiatry, University of Oxford, Oxford OX3 7JX, UK; af Division of Evolution, Infection and Genomic Sciences, Faculty of Biology Medicine and Health, School of Biological Sciences, The University of Manchester, Manchester, UK; ag Manchester Centre for Genomic Medicine, Manchester University NHS Foundation Trust, Manchester, UK; ah College of Medical Veterinary and Life Sciences, University of Glasgow, Scotland, UK; ai Royal (Dick) School of Veterinary Studies, The University of Edinburgh, UK; aj School of Life Sciences, Physiology, Pharmacology, and Neuroscience Division, Medical School, University of Nottingham, Nottingham NG7 2UH, UK; ak Department of Neuroinflammation, UCL Queen Square Institute of Neurology, London, UK; al Molecular and Clinical Sciences Research Institute, St George's University of London SW17 0RE, UK; am Department of Neurology, St George's University Hospitals NHS Foundation Trust, London, UK; an The Roslin Institute, Royal (Dick) School of Veterinary Studies, The University of Edinburgh, UK; ao UK Dementia Research Institute Care Research and Technology Centre, Imperial College London and the University of Surrey, UK; ap Department of Psychology, University of Illinois, Urbana-Champaign, Champaign, IL, USA; aq Department of Bioengineering, University of Illinois, Urbana-Champaign, Urbana, IL, USA; ar Beckman Institute for Advanced Science and Technology, University of Illinois, Urbana-Champaign, Urbana, IL, USA; as Department of Neuroscience, Physiology and Pharmacology, University College London, UK; at School of Psychology and Neuroscience, University of Glasgow, UK

**Keywords:** Vascular cognitive impairment, Vascular dementia, Small vessel disease, Cerebral vascular disease, Dementia

## Abstract

Although dementia research has been dominated by Alzheimer's disease (AD), most dementia in older people is now recognised to be due to mixed pathologies, usually combining vascular and AD brain pathology. Vascular cognitive impairment (VCI), which encompasses vascular dementia (VaD) is the second most common type of dementia. Models of VCI have been delayed by limited understanding of the underlying aetiology and pathogenesis. This review by a multidisciplinary, diverse (in terms of sex, geography and career stage), cross-institute team provides a perspective on limitations to current VCI models and recommendations for improving translation and reproducibility. We discuss reproducibility, clinical features of VCI and corresponding assessments in models, human pathology, bioinformatics approaches, and data sharing. We offer recommendations for future research, particularly focusing on small vessel disease as a main underpinning disorder.

## Highlights

• Different models recapitulate different aspects of human SVD – the right model should be used to answer the right question.
• Successful translation requires close-working relationship between preclinical and clinical researchers to identify bridging points linking the basic science to the clinics.
• Co-ordination of more rigorous, robust, and detailed preclinical evaluation of animal models is required through the concept of pRCTs.
• The UK DRI vascular Theme, alongside DPUK experimental medicine Incubator, are working towards building a community to boost VCI preclinical and clinical research.

## 1 Introduction

Dementia is a major global public health problem, with about 55 million people worldwide [1] thought to be living with dementia, although this figure may be an underestimate with dementia being under diagnosed, particularly where healthcare provision is thinly spread. Dementia prevention, identification and treatment is now a priority for many governments however research spend remains below that of other major non-communicable diseases [2].

Vascular cognitive impairment (VCI), which encompasses vascular dementia (VaD) is the second most common type of dementia, caused as a result of vascular injury to the brain [3]. Although dementia research has been dominated for decades by Alzheimer's disease (AD), most dementias in older people are now recognised to be due to mixed pathologies, usually combining vascular and AD brain pathology [4]. ‘Vascular contributions to cognitive impairment and dementia’ (VCID) is used when considering wider effects of vascular disease in mixed dementias, and with VCI, are now preferred terms to VaD.

Historically, VCI was considered to occur after a stroke and to have a step-like clinical course as new strokes occurred [5]. Stroke is a risk factor for dementia [6]. While VCI can result from haemorrhagic or ischaemic stroke, the commonest cause is now recognised to be subcortical microvascular disease also known as small vessel disease (SVD) [7].

Models of VCI, including SVD, have been delayed by limited understanding of the underlying aetiology and pathogenesis. To address this issue, in January 2017 we convened a workshop to discuss ‘Small vessels, dementia and chronic diseases – molecular mechanisms and pathophysiology’ [8], supported by Dementias Platform UK (DPUK-1), British Heart Foundation (BHF) and Royal Society of Edinburgh. This multidisciplinary workshop, and subsequent review paper [9], identified a range of potential models and mechanisms that mimicked some or all of the epidemiological or histopathological features of human SVD. The workshop also highlighted limitations and implications for future research that were necessary to bridge major gaps in knowledge [9]. Some of these were addressed in a subsequent meeting addressing assessment of cognition in preclinical models [10].

To assess progress in the field in modelling SVD and VCI, identify priorities for immediate future research, and recognising the major additional Government investments in dementia research in the UK, we reconvened the workshop in March 2022, organised and supported by UK Dementia Research Institute (UK DRI), DPUK-2 and BHF. The workshop brought together key experts from multidisciplinary, diverse (sex, geography and career stage), cross-institute groups, drawn from as many UK labs working on preclinical VCI and clinical experts as possible. It addressed important points on other vascular models, reproducibility, clinical features of VCI and corresponding assessments in models, human pathology, bioinformatics approaches, and data sharing. There were several focused break out discussions, with feedback and discussion by the whole group. In this report, we summarise the key points raised by experts and outputs of the focused group discussions, including recommendations for future research, particularly focusing on SVD as a main underpinning disorder.

## 2 What have we learned since the first workshop in 2017 [9]?

Since the workshop in 2017, there has been progress in understanding human SVD mechanisms [11,12] and symptoms [13], in harmonising methods to translate between preclinical and clinical SVDs studies [[11], [14]] and in reverse translation to unpick SVD mechanisms in preclinical models. For example, systematic reviews had identified Spontaneously Hypertensive Stroke Prone (SHRSP) rats as a potentially relevant rodent model of sporadic SVD [15,16]. The SHRSP model develops hypertension reliably in adolescence, superseded by endothelial cell (EC) autonomous dysfunction [17], rendering it vulnerable to vessel and tissue damage from hypertension in later life. This EC dysfunction includes impaired tight junction formation, impaired nitric oxide (NO) production, microglial activation and blocking of oligodendrocyte precursor (OPC) maturation [17]. This EC dysfunction has now been associated with gene *ATP11B* [17] and subsequent development of the *ATP11B* knockout rat showed the same cellular, histopathological and cognitive-behavioural abnormalities as the SHRSP, in the absence of hypertension [18]. This demonstrates that an EC autonomous dysfunction can cause SVD, without hypertension, consistent with clinical observations [[17], [18]], and clinical trial data showing that a) antihypertensive therapy, even intensive antihypertensive therapy, has modest effect on preventing SVD progression [19], and b) drugs which restore EC function (replace NO, unblock OPC maturation block) reduce recurrent stroke, cognitive impairment and dependency long term after small vessel (lacunar) stroke [20].

For monogenic SVDs, there are also more reliable models of CADASIL [21], COL4A1/COL4A2 [22,23], CARASIL [24], TREX [25]; and while each might start with a different gene-protein abnormality, the consequences at the glio-vascular unit and for the neuron, are similar – altered basement membranes, inflammation, impaired vascular function and secondary tissue damage. Additional models that explore effects of hypertension and diet in sporadic SVD have been developed [26].

As a further example, the carotid coil model, which is thought to mimic some brain microvascular and tissue changes of sporadic SVD via hypoperfusion [27], may instead be acting mainly through increased carotid (and intracranial) vascular stiffness, as shown by increasing data from human epidemiology studies [28]. Furthermore, an early event after coil application is short term blood brain barrier (BBB) leak [29], suggesting that ‘generic’ pathophysiological processes that damage vessels and tissue can arise from a range of triggers. However, many gaps remain (Table 1). The following sections describe the present Workshop participants’ proposals for translational approaches to accelerate from understanding to effective prevention and treatment of VCI.

**Table 1:** Gaps in knowledge and requirements to advance knowledge of human cerebral small vessel disease.

Feature	Requirements for early advances in knowledge	Gap in knowledge or resource
General	• Recognise that different models recapitulate different aspects of human SVD – use the right model in the right situation; • Core set of reference standard techniques for preclinical, neuropathological and clinical studies; • Models/cells reflect age, sex of human populations; • Access subcortical structures, rather than impute these from cortical results; • Longitudinal studies to older ages; • Comorbidities represented appropriately in models; • Should large mammals be used in specific model situations instead of rodents?	• Better model descriptions and look-up tables; • Better preclinical standards; • Improved neuropathology descriptors; • Improved harmonisation of human cognitive assessments; • Routine collection of markers of early life factors (education, socioeconomics, peak cognitive ability) • Adoption of human neuroimaging definitions and descriptors into preclinical research (e.g. STRIVE1, 2); • Preclinical MRI should obtain equivalent sequences to those used as standard in human studies

Vessels	• Variation in arteriole, capillary, venule anatomy across different brain regions and arterial territories; • Implications of morphological differences for vascular function in health and disease; • EC – cell-autonomous vs non-cell-autonomous disease and triggers; • Does EC dysfunction always affect oligodendrocyte precursor cell maturation, activate microglia, impair tight junction formation, affect astrocyte end feet?	• Methods to enrich tissue extracts for ECs; • Data on vessel morphology and function in different brain regions and tissues; • Use the retina routinely in rodent models (as in human SVDs) to visualise arteriolar/venular, retinopathic and nerve fibre layer changes to advance understanding of brain changes in rodent models

Vascular function	• Regional and tissue type variability in cellular and tissue vulnerability to altered cerebral blood flow, O2/metabolite delivery, waste clearance; • Influence of genetic and early life factors on tissue and cellular vulnerability to altered vascular, energy and waste clearance homoeostasis; • Does BBB permeability increase occur early in SVD pathogenesis? Does it fluctuate?	• Do all SVD model mechanisms show altered BBB, cerebrovascular reactivity (CVR), stiffness, i.e. the same vascular dysfunctions? • Does BBB permeability increase predate or occur simultaneously with impaired CVR or increased stiffness?

Glia–oligodendrocyte, astrocyte, microglia	• Is OPC maturation block a generic and universal consequence of EC dysfunction? • How does EC and/or vascular dysfunction affect astrocyte endfeet and neuronal energy transfer? • How do ECs and pericytes interact in health and disease? • Effect of early life factors on connectivity and myelination • How do microglia sense and signal to other vessel components? • What role do microglia responding to vessels damage/dysfunction play in the association of myeloid risk genes with dementia	• A reliable ‘neuro-glio-vascular unit on a chip’ or agglomerate that included vascular structures • Reliable multicell-type BBB models

Inflammation	• Endogenous or exogenous (systemic) or both? • Primary trigger or secondary consequence of vascular damage/dysfunction?	• Origin of inflammatory cells in the brain, in the perivascular space (PVS) in the vessel wall • Effect of systemic inflammatory triggers and cytokines • Effect of BBB leak on perivascular inflammation

Fluids, waste clearance	• Direction of fluid and solute travel in periarteriolar perivascular spaces – in or out of the brain? • If direction differs, is it generalised, regional, or vessel specific? • Does interstitial fluid exit via perivenous spaces? • Does perivenous space fluid mix with ‘clean’ cerebrospinal fluid (CSF) in the subarachnoid space or remain separate? • Main CSF/fluid exit routes from the cranium and proportions of fluid exiting via each route; • Role of vascular pulsation, respiratory motion, vasomotion in moving fluid through the cranium	• Studies addressing PVS in subcortical tissues that assess both CSF uptake and solute clearance (IPAD) in the same experiment; • Studies of PVS function in health and disease by age; • Studies of perivenous spaces in rodents; • Studies of large mammalian neurofluids systems; • Variation of fluid clearance with sleep/wake cycles; • Adverse effects of altered sleep on fluid clearance

Cognitive-behavioural relevance	• Characterise the cognitive and behavioural consequences of SVD in models, including at different stages	• Standardised cognitive and neurobehavioural tests that are relevant to rodent (or large mammalian) function without needing to be ‘learned’

Interventions	• Interrogate effects of repurposable drugs in models to identify promising agents to test in people; • Multicentre preclinical studies including randomised clinical trials, and platform trials	• Multicentre preclinical studies including randomised clinical trials, and platform trials • Ongoing continuously updated systematic reviews and meta-analyses of drug studies in preclinical models; • Continuously updated systematic reviews and meta-analyses of results of trials of potential agents to treat human SVDs

## 3 Co-morbidities, inflammation and cerebrovascular disease

### 3.1 Co-morbidities

**Fig. 1:**
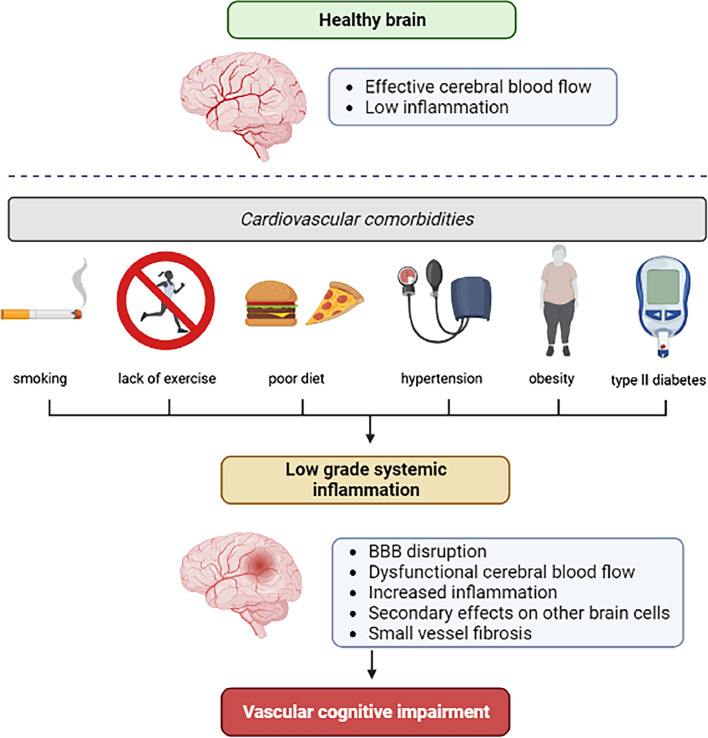
Hypothesised links between co-morbidities and vascular cognitive impairment, potentially mediated by low grade inflammation. Figure kindly provided by Josephine Thomas.

Most SVD is sporadic and commonly associated with comorbidities that are also vascular risk factors (e.g. hypertension, diabetes, stroke, AD; Fig. 1). This heterogeneity is not replicated in preclinical studies, and experimental models seldom include common risk factors for cerebrovascular disease and dementia [30], despite the fact that they clearly influence disease pathology [31]. Studies typically concentrate on modelling the genetic form of SVD such as CADASIL [21] or one potential aspect of sporadic SVD, such as hypertension [15], to enhance reproducibility in a controlled setting, rather than modelling diversity to improve translation*.* It is possible that sporadic SVD is a combination of comorbidities or environmental factors in addition to SVD-associated pathways or genes, which are below the threshold to develop SVD in isolation and therefore overlooked. Several rodent models are currently used in comorbidity research fields (see Table 2) [32,33] and there is the possibility of combining these in future research, although a consensus of which models are relevant for SVD and VCI will need to be reached.

**Table 2:** Rodent models used within the comorbidity research field.

co-morbidity	model	Refs.
Ageing	samp8 (senescence-accelerated mouse)	[34]
	Environmental stress models	[35]

Hypertension	SHRSP (spontaneously hypertensive stroke-prone rats)	[36]
	SHR (spontaneously hypertensive rats)	[37]
	Dahl salt-sensitive rats	[38]
	Angiotensin II-induced hypertension	[39]
	Salt diet-induced hypertension	[40]

Diabetes Mellitus/ Hyperglycaemia	*db/db* mice (obese type 2 diabetes mellitus)	[41]
	*ob/ob* mice (obese type 2 diabetes mellitus)	[41]
	Zucker rats (obese type 2 diabetes mellitus)	[42]
	Goto-Kakizaki Rat (non-obese type 2 diabetes mellitus)	[43]
	Streptozotocin- induced type 1 diabetes mellitus	[44]
	High-fat diet induced obesity	[45]

Hyperlipidaemia	ApoE KO (apolipoprotein E knock-out) mice	[46]
	Low-density lipoprotein receptor (*Ldlr−/−*) knockout mice	[47]

The importance of multimorbidities typical of ageing or lifestyle factors is illustrated by the common co-occurrence of multimorbidities and cognitive decline and the increasing epidemiological evidence suggesting that older adults who maintain an active lifestyle involving a healthy diet, mental, social and physical activities are protected, to a certain degree, against cognitive decline or dementia [48]. The European Stroke Organisation (ESO) Guideline Working Group on covert SVD found few randomised trials but strong observational evidence to support adoption of a healthy lifestyle including diet, exercise, avoidance of smoking, and control of hypertension to prevent progression of covert SVD into clinical outcomes of stroke and dementia [19]. Although these largely observational findings have yet to be translated into strong evidence, nonetheless they are sensible public health measures and support the inclusion of co-morbidities in the design of rodent models of SVD and VCI.

### 3.2 Inflammation

Both systemic and peripheral inflammation are recognised as an important contributors to the pathophysiology and outcome of stroke and SVD [[49], [50], [51]], although whether they are causal or secondary to the disease process still remains to be determined. Sources of inflammation that affect the brain are not restricted to hallmark neuroinflammatory changes in the brain, such as certain forms of microglial and astrocyte reactivity, BBB breakdown and leucocyte recruitment, but also include systemic inflammatory disorders [52]. Stroke is well known to provoke systemic inflammatory responses which correlate with stroke severity [53] and in turn the risk of post stroke cognitive impairment, and SVD has been associated with reprogramming of the peripheral immune system into a proinflammatory state [54,55]. Further, in both animal models and humans, common vascular risk factors can lead to vascular neuroinflammation, and eventually neuroinflammation (Fig. 1) [56].

Thrombo-inflammation refers to the contribution of platelets and coagulation pathways to disease, and is important in stroke [57]. It is much less studied in dementia, though recent studies suggest a role for activation of the VWF/ADAMTS13 (von Willebrand factor/ADAM metallopeptidase with thrombospondin type 1 motif 13) axis [58,59]. A novel constitutively active variant of ADAMTS13 was recently reported in acute stroke [60], and can be used as a tool model, alongside other sophisticated tools, such as biodegradable and ultrasensitive microprobes to track immune response [61] and investigate the contribution of inflammation in models of VCI.

## 4 Translating model data to human tissues - The Neuropathological viewpoint

**Fig. 2:**
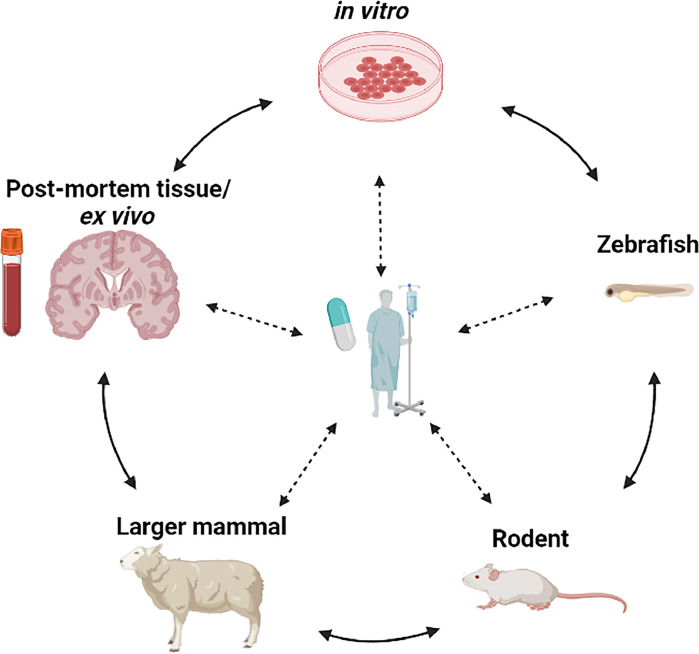
Bi-directional translation within the clinical and preclinical fields are essential in furthering our understanding of VCI. Figure adapted from [62], and kindly provided by Josephine Thomas. [62].

There are fundamental differences between rodents and humans that limit the formers’ relevance to understanding of human disease pathophysiology. If animal models are to play an important role in pathophysiological discovery, then they need to replicate features and underlying causal mechanisms seen in the human brain, from the primary vascular changes through to the secondary parenchymal changes, highlighting the exceptional importance of close-working between preclinical and clinical researchers in SVD, to drive relevant bi-directional translation (Fig. 2).

**Fig. 3:**
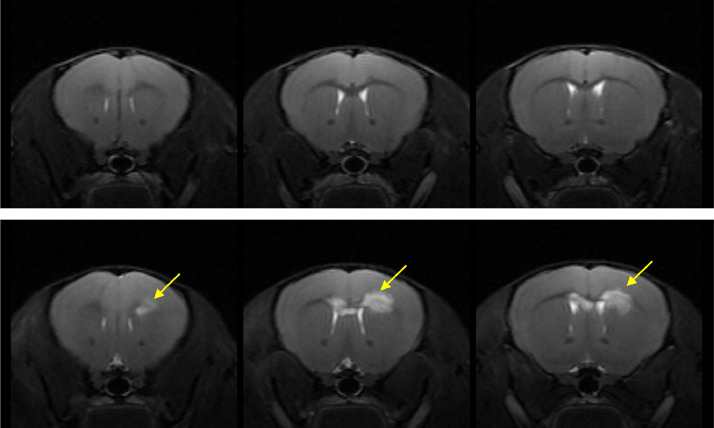
T_2_-weighted images from mice undergoing bilateral carotid artery stenosis (BCAS) induced hypoperfusion, the bottom panel highlights white matter lesions (yellow arrows). Figure kindly provided by Dr Tracy D Farr.

One of the hallmarks of SVD is the presence of diffuse white matter lesions, seen as white matter hyperintensities (WMH). There are distinct differences between the structures of a rodent and a human brain, including gyrencephalic versus lissencephalic cortical structure and differences in the organisation of subcortical regions [63,64]. Further, while it is possible to observe acute ischaemic lesions in the white matter of rodents (Fig. 3), diffuse white matter changes resembling clinical WMH are not commonly observed in rodents [65]. Recent advances in the regional mapping of rodent and human brains, by comparative transcriptomic [66] and functional methods [67], can be used to systematically understand the limitations of the rodent brain to avoid mis-interpretation of preclinical research.

Though mice are more generally used for genetic manipulation studies, there are recent transgenic rat models [[18], [68]]. Rats offer some advantages over mice for behavioural testing and for white matter MRI [[10], [69]]. Large experimental species (primates, canines, sheep etc.) offer more human-like brain structure, with extensive white matter. These are not amenable to high volume drug screening. Rather they are likely to be of value in mechanistic studies and focused dose finding studies prior to human use [70].

**Table:** Panel 1 - Neuropathologist's viewpoint of *in vivo* models

Be clear about what the model is actually modelling. What aspect of the human spectrum of SVD is being assessed?
Where possible, compare or relate animal model tissue-level observations to human tissues. Is the animal model observation relevant to the human disease?
SVD – even quite severe SVD – can be clinically silent
A “good” model need not have cognitive phenotypes
A SVD model needs some vascular pathology

## 5 Translating animal model data to clinical trials - The clinical viewpoint

**Fig. 4:**
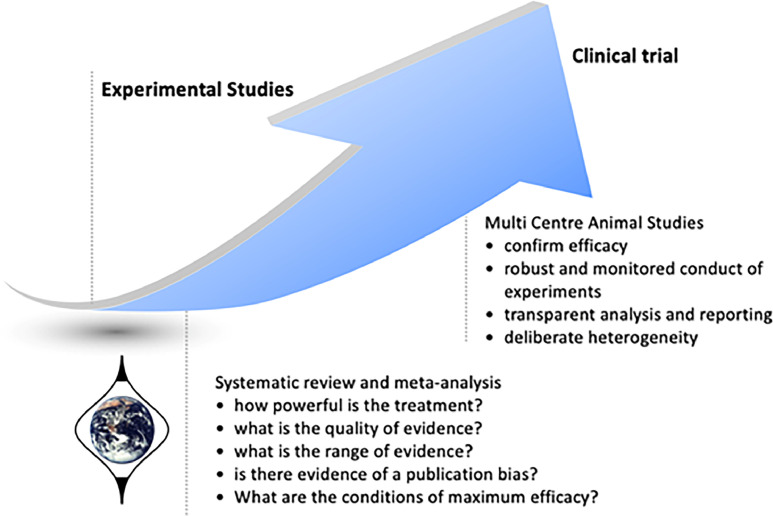
Evidence based translational medicine using results of systematic reviews and meta-analysis to drive multicentre animal studies. Figure kindly provided by Prof Malcolm Macleod.

Multi-centre randomized controlled trials (RCTs) are the standard for clinical evidence on therapies, often involving large sample sizes at phase 3 to increase generalisability. In the case of preclinical studies, multicentre approaches using methods adapted from clinical trials (Fig. 4), such as Multi-PART (Multicentre Preclinical Animal Research Team; https://cordis.europa.eu/project/id/603043/reporting), could help overcome poor inter-laboratory replication.

Another methodological advance that has not yet been fully implemented in the clinical dementia field is the multi-arm, multi-stage (MAMS) trials platform [71] widely used in cancer, and recently in COVID-19 [72]. This type of ‘rolling’ trial avoids a number of pitfalls associated with ‘single-use’ protocols, enabling faster testing, and where appropriate, rejection of interventions. A number of trial platforms have been developed for testing interventions in dementia [73], although to-date these have been limited to pre-symptomatic prevention trials, or rarer genetic forms of dementia [74,75].

Over a decade ago a group of stroke researchers proposed a multicentre, randomised and blinded preclinical trial (pRCT) to improve translation for novel therapeutics [76]. Such a trial would not replace the curiosity-driven preclinical research which identifies and validates a therapeutic target. Rather, it would be an additional step prior to clinical testing [77]. Such trials are logistically complex, requiring large-scale funding, intense oversight by a steering group and multiple ethical approvals beyond those required for individual research studies. Nevertheless, some have been successfully completed and reported [78,79], most recently, the NIH funded the Stroke Preclinical Assessment Network (SPAN). The aim of SPAN is to conduct a randomized, placebo-controlled, blinded, multi-laboratory trial using a MAMS protocol to identify one or more potential stroke treatments with a high chance of success in human clinical stroke trials [80], recently completing a proof of concept trial that assessed several acute stroke treatments [81]. We could learn from the success of the stroke field by co-ordinating more rigorous, robust, and detailed preclinical evaluation within the VCI field through the concept of pRCTs using MAMS protocols.

## 6 Current models and how to improve them: consensus from group discussion

A driving factor for the shortfall of translatable interventions in dementia research is uncertainty over disease models that can achieve this, driven by our limited understanding of the causes and progression of VCI. Instead, we could aim to capture key features that accurately reflect clinical SVD. Although the models might potentially only capture one relevant feature or process, a focus on replicating the process as accurately as possible could increase the relevance of the model. To achieve this, clear communication between preclinical and clinical fields is required to identify which features should be modelled and how best to measure them. Table 1 summarises Gaps in Knowledge and requirements to advance knowledge in human VCI and SVD, updated from the first workshop. The following headings were discussed by a multi-disciplinary roundtable, with a range of clinical and non-clinical expertise at all levels of seniority from graduate students through to senior Investigators.

### 6.1 Limitations of rodent models

Rodent research has its disadvantages (Table 3). There are substantial costs for generating a surgical model or transgenic strain. Furthermore, longitudinal studies often lead to survivor bias or a lack of sufficient power as rodents that display stronger phenotypes may not achieve the most chronic endpoint. Rodent development and ageing follows a different time-course than humans, and this must be considered during experimental design. In most experimental settings, animals have a sedentary life, unlimited access to food, are protected from pathogens and other environmental stresses, and this may also influence ageing. How the processes that underlie human ageing can be better modelled in rodents is highly debated. For example, genetically altered (progeroid) mouse models display premature ageing due to mutations in ageing-related genes, though their relation to typical ageing in humans is unclear [82]. Models of accelerated senescence have also been developed [83,84] as well as environmental stress models (ozone and radiation exposure) that also display features of accelerated ageing and frailty [35].

**Table 3:** Strengths and limitations of preclinical models used within the VCI field.

Model	Strengths	Limitations
iPSC	• Human model system • Genetic diversity • Ease of genetic manipulation • High throughput drug screening and toxicity studies	• Constrained artificial environment • Lack complex tissue organisation and physiological context • Quality, purity and maturity of differentiated cells • Significant variability in differentiation potential and genetic stability between iPSC line • Absence of vascularization • Lack cognitive outcome measures

Organoid	• Human model system • Spatial organization of tissues, cell-cell and cell-matrix connections • Model complex interaction and connection amongst brain regions and structures • More mature phenotype of iPSC-derived cells • Can be maintained for extended periods • Drug screening and toxicity studies	• Lack complex organisation of the *in vivo* brain • Significant variability in differentiation potential and genetic stability between iPSC line • Absence of vascularization • Absence of microglia • Lack of cognitive outcome measures

Zebrafish model	• Ease of genetic manipulation • Transparent during development allowing for non-invasive *in vivo* imaging • Prolific reproduction rates • Basic functional outcome measures • Drug screening and toxicity studies	• Simpler nervous systems • Genomic differences between zebrafish and human greater than mammalian models • Lack higher cognitive outcome measures

Rodent models	• Study of non–cell-autonomous effects • Rapid assessment of neuronal and circuit function • Cognitive outcome measures • Availability of powerful genetic toolkits • Greater acceptability in terms of ethics compared to large mammalian model	• Small white matter volume • Different brain structure relative to human • Domain specific genomic differences between rodent and human • Species differences when evaluating cognitive deficits and their relevance to human SVD progression • Inbred animals do not reflect the genetic diversity of a population • Short lifespan of rodents means that it is difficult to reproduce the symptoms of dementia

Large mammalian model	• Gyrencephalic brain anatomy • More white matter • More human-like vasculature • Longer lifespan than rodents • Non-human primate models allow for sophisticated cognitive tests and have a very close evolutionary relationship to humans	• Relative lack of behavioural assays currently available compared to rodent models • Scarcity of species-specific reagents • Costly and therefore constrained by the number of centres which have the infrastructure and resources to house this model • Longer duration of studies • Ethical constraints

The value and interpretation of behavioural testing in rodents needs to be considered. While a composite of tests is often used in humans, equivalent tests for rodents should be appropriate to rodent behaviour, function and cognition and not require months of training or food restriction which may confound the mechanism of interest. Moreover, task-relevant sensory, motor and anxiety confounds of behavioural performance should be analysed whenever possible to ensure that poor task performance is not misinterpreted.

### 6.2 Alternatives to rodent models

There are a variety of *in vitro* platforms that complement *in vivo* research (Table 3) and hold promise to replace animals in the future, such as cell cultures derived from induced pluripotent stem cells (iPSCs). Somatic cells can be derived from patients with a genetic predisposition and dedifferentiated to form iPSCs, which can be further differentiated into multiple cell types, for instance into the different cell types in the neurovascular unit, or into organoids or agglomerates [85,86]. This facilitates study of biological processes, such as maintenance of BBB, extracellular matrix maintenance and immune cell signalling within the context of the genetic predisposition or risk factor but within a highly constrained environment. These cell culture platforms can also be used to screen large numbers of drugs, prior to *in vivo* testing and recent developments such as CRISPR (Clustered Regulatory Interspaced Short Palindromic Repeats) editing can facilitate additional manipulations. However, their limitations are important: the constrained environment within the cell preparation that may lack diversity in the native cell population, the artificial environment in relation to the integrated physiology of the whole organ or animal, the lack of vasculature, and these developmental cells could have limitations when modelling age associated diseases, or anything resembling cognitive outcome measures.

Other animal models are also currently being explored (Table 3). Zebrafish have the advantages of prolific reproduction rates and larval transparency allowing for live imaging, coupled with numerous genetic reporter lines [87]. Higher in the evolutionary tree, larger mammals (e.g. sheep, dogs, pigs or primates) have more white matter, closer in proportion and structure to that seen in humans, and more human-like vasculature [[88], [89], [90], [91]]. An interesting alternative approach to laboratory studies is the use of companion animals (dogs, felines) for studies of common disorders like VCI or SVD, including relevant lifestyles, and assessment of animal behaviour, cognition and brain pathology.

### 6.3 Bridging points between preclinical and clinical studies

Successful translation of preclinical studies requires bridging points linking the basic science to the clinics (Fig. 2). For example, Magnetic Resonance Imaging (MRI) can be performed in animals, using equivalent sequences as in clinical scans, and thus provide translational information on structural changes and vascular function [14]. Therefore findings in both species, such as enlarged perivascular spaces [92], dysfunctional BBB or cerebrovascular reactivity can be compared and provide reassurance that the model or intervention is relevant to human disease [14]. Similarly, molecular and cellular level association between the disease model and human disease through, for example, -omics-based cell profiling and fluid biomarker measures would enable fairly objective “species-bridging” measures.

Cognitive function in patients can be assessed with multiple tasks covering a large range of cognitive domains. Rodent behaviour is well understood but requires more research to develop tests of cognition that are relevant to VCI by mapping onto human cognitive domains affected in VCI [[10], [93]]. A UK consensus on assessment in preclinical studies of VCI has already been published and should be more widely followed [10].

There are a number of innovations from research in other conditions that may potentially transform how we design future trials in the field of VCI. Current dementia trials still rely on relatively dated outcome measures, such as ADAS-Cog (Alzheimer's Disease Assessment Scale, cognitive subscale) and CDR (Clinical Dementia Rating) [94], which are often performed at infrequent intervals. Wearable technologies and other technical devices make it increasingly possible for researchers to access granular information about daily activities, from walking and sleeping to device interaction (e.g. sleep mat to monitor sleep patterns, gait speed and laterality monitoring devices). These can potentially provide a far more thorough understanding of treatment effect, as well as allowing for better detection of adverse events and side effects [95]. Such detailed datasets can also potentially be combined with a ‘n of 1′ approach, allowing researchers to evaluate the effects of interventions on an individual basis [96]. By analogy, outcomes in preclinical studies should aim to capture cognition, function, mobility and activities, for example via 24/7 cage-monitoring technology [97], to provide a more comprehensive profile of the animal's status.

### 6.4 Bedside-to-bench approach

In stroke, most current treatments were developed through clinical research testing drugs repurposed from other vascular disease – e.g. aspirin for secondary prevention, thrombolytic agents to remove occlusive thrombus –not from drugs or mechanisms identified in preclinical models. The pharmaceutical industry was subsequently able to develop more effective antiplatelet (e.g. Clopidogrel) and thrombolytic agents (e.g. Alteplase, Tenecteplase) following testing in preclinical models. This contrasts with the perceived ‘conventional’ route by which drugs are developed and tested from research at the ‘bench’ and translated to the ‘bedside’.

This ‘bedside-to-bench’ approach could work well in VCI by testing repurposed drugs from other diseases that have potentially relevant modes of action on the proposed mechanisms in SVD. This repurposing approach is not commonly followed, especially by the pharmaceutical industry, and existing viable drugs might be dismissed [98]. Preclinical ‘platform’ trials, including MAMS trials described earlier, would offer a valuable complementary approach to drug testing to help determine potential modes of action of repurposed drugs that showed promise in clinical trials, and could help design better compounds.

The process of bedside-to-bench can also be informed by analyses of electronic health records. This approach was used for COVID-19 by the UK Longitudinal Linkage Collaboration (https://ukllc.ac.uk/) and in AD [99]. We can also use large longitudinal research registry datasets for VCI research, associating the outcomes of the diseases for at-risk individuals with potential factors such as lifestyle, risk factors or medication (see Panel 2 for useful resources). However, caution is required when interpreting effects of medication in electronic health records or research registry data since the allocation of medication is not randomised and many sources of bias are likely to exist in the data. However, the findings might broaden the understanding of the disease and reveal potential (alternative) therapeutic targets that have been overlooked by the conventional view.

Moving forward, better links between academia and industry, including large pharmaceutical companies, small and medium enterprises, contract research organisations, and start-ups, will facilitate multicentre collaborations and more rapid progression in finding new treatments for VCI. Finally, ‘industry bootcamps’ would educate academics on how to approach industry with an idea, how to put together a research package to present to industry, and how to start and maintain a mutually beneficially relationship with industry stakeholders.

### 6.5 Improving standardisation and reporting of data

There is a growing requirement to standardise research through reproducible protocols and standardisation between models. Lessons can be learnt from the success of the preclinical stroke field, that has come a long way in the pathophysiological understanding of stroke. Over the last twenty years, attempts have been made to refine experimental methods used in preclinical stroke research, improve reproducibility and reduce the number of animals used largely via the publication of guidelines. The best-known guidelines in preclinical stroke research are the Stroke Therapy Academic Industry Roundtable (STAIR) guidelines [100,101]. Further guidelines are aimed at the use of stem cells in preclinical stroke models [102], methodological approaches to improve animal welfare and scientific outcomes via the IMPROVE (Ischaemia Models: Procedural Refinements Of *in vivo* Experiments) guidelines [103] and merging of previously published guidelines into a more rigorous approach via the RIGOUR guidelines [104]. The same principles can apply to the VCI field, including having a central database of standardised protocols for behavioural testing, surgical procedure, and *ex vivo* experiments which would permit consistency of protocols across institutes, and facilitate meta-analyses.

To address the issue of transparent reporting, and facilitate reproducibility, the ARRIVE criteria (Animal Research: Reporting of *in vivo* Experiments) were published in 2010 [105] and updated in 2020 [106]. This includes careful definition of the independent experimental unit in the study (e.g. the animal/cage) and the study design including the control groups included. Defining the sample size required for the principal outcome measures prior to the experiment, using experimentally determined standard deviations and effect sizes to ensure sufficient experimental power whilst minimising the number of animals in the study. Ensuring that randomisation and blinding are used during both data acquisition and analysis avoids bias. Full reporting of the model used to include recognised nomenclatures and reference numbers, age, sex, experimental procedures, husbandry conditions and all other associated-meta- data is also critical. As well as the reporting of drop-out and any exclusion criteria (e.g. because of technical failure or welfare issue) and full reporting and justification of statistical analysis methods. Pre-registration of the study protocol including the above points improves research reliability. Ensuring complete adherence to the ARRIVE 2.0 essential 10 checklist will significantly enhance the translational value of preclinical research and researchers are encouraged to use them to increase the benefit of their research output and its long-time impact on patients.

Alongside guidelines for conducting and reporting preclinical research, a number of initiatives from the wider biosciences community including clinical research may serve to promote reproducibility, including open access practices [107], study preregistration [108] and resources to improve experimental design and analysis [109]. Within clinical research it is routine to conduct a systematic review to assess treatment effectiveness, and to routinely publish negative or neutral studies, however both are less common practices in preclinical research. Systematic reviews are an essential tool for obtaining an objective view of all the available evidence on a topic (thus helping to avoid repeating research that does not need to be repeated), and identifying potential disease mechanisms, or therapeutic targets for further investigation in larger, even multicentre*, in vivo* studies, prior to clinical testing. This approach has been highly effective in identifying (and excluding) potential SVD models [[15], [16], [110]], SVD pathology [111,112], and potential drugs to treat SVD in preclinical studies [113] and clinical trials [[20], [98], [114]], leading to promising results improving outcomes in SVD [20]. The extent to which the results of systematic reviews might be biased due to missing unpublished negative or neutral studies can be assessed through techniques such as funnel plots and by approaching authors for unpublished data, and should not preclude the use of systematic reviews as a highly valuable research tool when conducted properly.

Whilst academia benefits from an environment that allows freedom of thought, a lot can be learnt from the ‘fail-fast’ industry approach. The industry model is designed to rapidly test reproducibility and validity, with no negative implications for failed compounds or targets. A shift in culture is needed toward reporting on approaches that lack efficacy, and to know when to abandon them rather than continuing a flawed premise or pathway.

### 6.6 Need for wider multidisciplinary approaches

A key strategy to accelerate the field could be to diversify interdisciplinary collaboration to areas not typically involved in vascular or neurodegenerative brain research. For example, mathematicians and informaticians can model animal and human neurovasculature and blood flow, which may provide insights into disease mechanisms. Furthermore, engineers and physicists are essential to develop novel MRI and microscopic imaging techniques, alongside computational neuroimaging [115]. An additional benefit of utilising expertise from non-traditional biological backgrounds, is that they typically do not require animal models and therefore support the 3Rs mission of replacement, reduction and refinement of animals used in research [116].

### 6.7 Building a SVD community forum

Perhaps a disconnect between clinical and preclinical research in SVD and VCI is contributing to the failure to translate between ‘bench and bedside’. It would help to share practical expertise (Standard Operating Protocols and experiences) as well as fundamental knowledge and standardised definitions of preclinical and clinical terminology. Efforts in this direction are now being implemented in the UK through the UK DRI Vascular Theme and DPUK Experimental Medicine Incubator, plus BHF research initiatives and regional clinically-orientated brain health initiatives. Furthermore, local research-to-clinic initiatives such as the Geoffrey Jefferson Brain Research Centre in Manchester (https://www.gjbrc.org) and the Row Fogo Centre for Research into Ageing and the Brain in Edinburgh (https://www.ed.ac.uk/clinical-brain-sciences/research/row-fogo-centre/about) are providing hubs of researchers to boost activity and awareness in the UK. The ESO Guidelines on SVD, part 1 Covert SVD [19] and part 2 Lacunar Ischaemic Stroke (in prep, publication expected autumn 2023), are providing a much needed benchmark to guide current best clinical practice. The nascent SVDs Clinical Services Collaboration will improve clinical services for patients with SVD as well as research infrastructure. The NIH-funded MarkVCID (Biomarkers for Vascular Contributions to Cognitive Impairment and Dementia) initiative in the USA has given a major boost to VCI and SVDs preclinical and clinical research and awareness of the condition in the USA – a similar national initiative would greatly accelerate research and improved clinical services in the UK.

There is a growing need for a centralised database of information on SVD models. For example, one such database is Alzheimer Research Forum (https://www.alzforum.org/), an online community resource of specific knowledge to promote communication, research, collaborative and multidisciplinary interactions [117]. No such database existed for SVD/VCI at the time of the workshop, but has now been started by the UK DRI (see Panel 2). It so far includes 14 models, and will become a very valuable resource for research into vascular contributions to neurodegeneration. Interested researchers are invited to submit data on animals not yet represented in the database (contact Sarmi Sri, s.sri@ukdri.ucl.ac.uk).

## 7 Summary

The UK DRI-DPUK-BHF workshop provided an opportunity to share knowledge, technical skills, facility access, funding opportunities and create collaborations. The establishment of vascular disease and dementia consortia, both nationally and internationally, needs to be community-driven and include researchers from different centres, disciplines, and backgrounds. Inclusion of ECR days to consortium meetings cultivates the next generation of VCI researchers, and has been promoted in the UK by the UK DRI Vascular Theme for all interested ECRs. Panel 2 highlights some important resources for researchers within the UK vascular community.

**Table:** Panel 2 – Useful resources to highlight to the VCI community

UK DRI Vascular theme (https://ukdri.ac.uk/research-themes)
DPUK portal (https://portal.dementiasplatform.uk/)
Vascular ECR community (https://ukdri.ac.uk/news-and-events/from-bench-to-bedside-bridging-the-gap-between-discovery-research-and-the-clinic-in-vascular-research)
Vascular models database (to be launched early 2024)
VISTA Cognition (https://www.virtualtrialsarchives.org/vista-cognition/)
StrokeCOG consortium (http://www.strokecog.ie/)
MultiPART (Multicentre Preclinical Animal Research Team) (https://cordis.europa.eu/project/id/603043/reporting)

## Declaration of Competing Interest

No conflict of Interest.
